# Preliminary evaluation of image quality in supine breast MRI using a dedicated wearable coil and accelerometer-based motion correction

**DOI:** 10.1186/s41747-025-00662-2

**Published:** 2025-12-22

**Authors:** Alexandre Megel, Karyna Isaieva, Lena Nohava, Freddy Odille, Marc Fauvel, Barbara Fischer, Elmar Laistler, Jacques Felblinger, Philippe Henrot

**Affiliations:** 1 Service de radiologie, Institut de Cancérologie de Lorraine Alexis Vautrin, Vandoeuvre-les-Nancy, France; 2 Université de Lorraine, INSERM U1254, IADI, Nancy, France; 3 High Field MR Center, Center for Medical Physics and Biomedical Engineering, Medical University of Vienna, Vienna, Austria; 4 Christian Doppler Laboratory for Patient-Centered Breast Imaging, Medical University of Vienna, Vienna, Austria; 5 CIC-IT 1433, Université de Lorraine, INSERM, CHRU de Nancy, Nancy, France

**Keywords:** Artifacts, Breast, Image quality, Magnetic resonance imaging, Patient’s position

## Abstract

**Abstract:**

Supine breast MRI offers easier comparison with other screening methods than the prone MRI, but is often impaired by respiratory artifacts. This study aims to identify a suitable acceleration technique for motion-corrected supine breast MRI with a dedicated wearable coil (BraCoil), and to assess the quality of the resulting images. This prospective, single-center study included ten healthy female volunteers. Supine breast MRI was acquired using the BraCoil equipped with seven accelerometers. The acquisition protocol included unenhanced T1- and T2-weighted sequences; 2-, 3-, and 4-fold acceleration, as well as “elliptical scanning”, were tested for T1-weighted images. Image reconstruction was performed using two methods: the manufacturer’s standard algorithm and an advanced nonrigid motion correction algorithm. The images were compared to each other and to conventional prone MRI via radiological scoring. Additionally, comparisons were made with supine breast MRI obtained using a multi-purpose torso coil reported in previous studies. This study showed that using 2-fold acceleration was optimal for motion-corrected supine breast MRI using the BraCoil. It also demonstrated that enabling the “elliptical scanning” would shorten the measurement time by 33% without compromising image quality. Finally, for the data acquired, the BraCoil provides superior motion-corrected image quality compared to the torso coil.

**Key Points:**

- BraCoil image quality outperforms the body coil for both uncorrected and motion-corrected images in supine acquisition for T1w and T2w sequences.
- Enabling the “elliptical scanning” in T1-weighted sequences does not impair image quality while shortening the measurement time by 33% (compared to the same sequence without enabling this option).
- A 2-fold acceleration for the T1-weighted sequence provides sufficient scan time reduction, whereas higher acceleration would lead to even shorter scan time at the cost of significantly lower image quality.

**Graphical Abstract:**

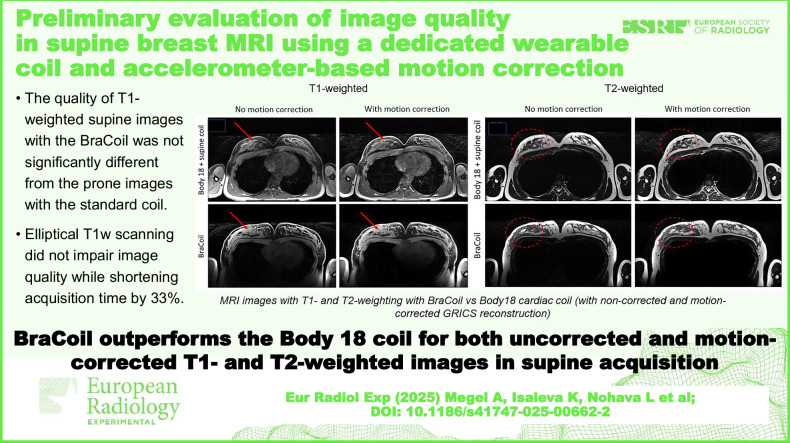

## Background

Breast cancer, the most common cancer in women worldwide, poses a substantial public health challenge owing to its morbidity and mortality [1]. Magnetic resonance imaging (MRI), typically performed in the prone position, is highly sensitive and specific for diagnosis [2–7], especially in younger women with dense breast tissue that may obscure mammographic results [8]. However, the prone position can cause discomfort, leading to anxiety or refusal of the exam [9], and may result in discrepancies between tumor location on MRI and during supine surgery, potentially affecting surgical outcomes [10].

A prototype wearable coil vest that allows MRI in the supine position (the BraCoil) could represent a major advancement in this field [11]. This approach is expected to better align diagnostic imaging with surgical positioning, thereby improving diagnostic accuracy and facilitating surgical planning [10]. The introduction of non-rigid motion correction techniques, such as “Generalized Reconstruction by Inversion of Coupled Systems” (GRICS) [12], as demonstrated in previous studies using a standard cardiac coil, allowed reducing motion artifacts caused by breathing, thus improving the quality of images in supine MRI [13, 14].

This study integrates the BraCoil with GRICS motion correction, aiming to determine an effective acceleration method optimized for this particular combination of techniques for supine breast MRI. It further seeks to evaluate the quality of the resulting images and compare them with those obtained using earlier supine breast MRI approaches.

## Materials and methods

### Population

This is a prospective, single-center study involving ten healthy female volunteers with no known history of breast pathology, aged between 29 and 52 years (38 ± 7 years). Breast density assessed according to the BI-RADS classification [15] revealed no mostly fatty breasts (category *a*), five cases of scattered fibroglandular density (*b*), three cases of heterogeneously dense breast (*c*), one case of extremely dense breasts, while one woman had silicone implants.

Written informed consent was obtained from each volunteer. Data were collected under the approved “EDEN” ethical protocol (ClinicalTrials.gov Identifier: NCT05218460).

### Data acquisition and correction

Images were acquired on a 3-T Prisma equipment (Siemens Healthineers, Erlangen, Germany), including T1-weighted fast low-angle shot and T2-weighted turbo spin-echo sequences, in two different positions: prone with the standard 18-channel breast coil and supine with the 28-channel BraCoil [11]. In the supine position, the arms were positioned alongside the body, and the BraCoil was positioned directly on the patient’s chest.

In the prone position, 80 slices with a thickness of 2 mm were acquired with a repetition time of 6,860 ms and an echo time of 97 ms for the 3-fold accelerated T2-weighted sequence. In the supine position, the T2-weighted images were acquired with 60 axial slices, 3-mm thickness, repetition time 4,880 ms, echo-time 100 ms, without acceleration, to enable motion-corrected (MoCo) reconstruction while keeping acquisition time short enough, and to consider the breast shape difference. The T1-weighted sequence was acquired with a repetition time of 4.14 ms, echo time of 1.84 ms, 192 axial slices, and slice thickness of 1 mm. In the prone position, a 3-fold acceleration in the left-right direction was applied. For the supine position, the T1-weighted sequence was launched three times, with three different acceleration factors: 2, 3, and 4. Additionally, the supine T1-weighted data with “elliptical scanning” were generated by retrospectively applying an elliptical k-space filter. This filtering was performed directly on the raw k-space data by setting to zero all peripheral lines lying outside the ellipse inscribed in the k_*y*_–k_*z*_ plane, while maintaining full sampling along the readout (k_*x*_) direction. If applied prospectively, “elliptical scanning” allows for a considerable reduction in measurement time. Scan times with and without elliptical scanning were predicted by the MRI system and validated using phantom acquisitions.

Seven MRI-compatible accelerometers (MARMOT [16]) were placed on the BraCoil to record the respiratory data. These data were then utilized to feed the GRICS algorithm [12] and enable it to correct motion artifacts as described by Isaieva et al [17].

### Image analysis

Images were independently scored by two radiologists. The junior radiologist (R1) had 4 years of experience in radiology and 1 year of experience in breast imaging, and the senior radiologist (R2) had over 25 years of experience in breast imaging. The data to be analyzed were randomized using a specialized plug-in of ArchiMed software [18], and the radiologists were blind to the participant identification, acquisition, and type of reconstruction.

Initially, a randomized subset of three participants was presented to the radiologists for training and consensus for each series. Then, all 10 participants were evaluated independently by each radiologist using a different randomization sequence.

Image quality was scored according for the following parameters: sharpness (1 = major blurring rendering the examination uninterpretable, 2 = significant blurring degrading interpretation, 3 = significant blurring allowing interpretation, 4 = minor blurring, and 5 = no blurring); uniformity (1 = significant signal inhomogeneity rendering examination uninterpretable, 2 = significant signal inhomogeneity degrading interpretation, 3 = signal inhomogeneity enabling interpretation, 4 = minor signal inhomogeneity, and 5 = no inhomogeneity); and overall quality (1 = very poor, 2 = poor, 3 = average, 4 = good, and 5 = excellent). For motion, folding, and chemical shift artifacts, scores were defined as follows: 1 = major artifact rendering examination uninterpretable, 2 = artifact compromising interpretation, 3 = significant artifact but interpretation possible, 4 = minor artifact, and 5 = no artifact.

Acceleration factors 2, 3, and 4 of the T1-weighted supine images were compared in terms of “rank”, “overall quality”, “motion artifacts”, and “folding artifacts”. The three datasets with different acceleration factors were simultaneously presented to the radiologists in randomized order. The optimal acceleration factor was defined as follows: if no significant difference was observed, the shortest acquisition was retained; if a significant difference existed, the best-quality images were retained.

“Rank” and “overall quality” were assessed with and without retrospectively applied “elliptical scanning” for T1-weighted supine images. Two images, corresponding to the two scanning types, were simultaneously shown to the radiologists in randomized order. The optimal option was defined as follows: if no significant difference was found, elliptical scanning was retained; if full sampling produced significantly better images, it was retained.

To compare image quality between prone images acquired with the standard breast coil and supine images acquired with the BraCoil, “sharpness”, “uniformity”, “overall quality”, “folding artifacts”, “motion artifacts”, and “chemical shift artifacts” were assessed using both uncorrected and GRICS MoCo supine images. Images were presented to the radiologists one dataset at a time, without specifying their acquisition and reconstruction type.

In addition, the data acquired using the BraCoil for supine MRI were compared to those of two previous studies by Isaieva et al [14] and Meullenet et al [13], which used the Body18 torso coil (Siemens, Erlangen, Germany). “Overall Quality” was selected as the single metric for this analysis. To eliminate inter-reader bias, only the scores from the senior radiologist (R2), who participated in all three studies, were included. However, differences in the datasets across the three studies mean that variations in scores may reflect differences in the population rather than the technique. Additionally, the considerable time elapsed between studies could influence the senior radiologist’s scoring. To address these sources of bias, we introduced a normalized score inspired by the Symmetrized Percent Change [19]:

$${\Delta }_{{{\rm{relative}}}}=100 \% \cdot2\cdot\frac{{{{\rm{Score}}}}_{{{\rm{prone}}}}-{{{\rm{Score}}}}_{{{\rm{supine}}}}}{{{{\rm{Score}}}}_{{{\rm{prone}}}}+{{{\rm{Score}}}}_{{{\rm{supine}}}}}$$

This metric quantifies the quality gap between supine and conventional prone images, independent of population differences. A smaller or negative relative difference indicates better image quality.

### Statistical analysis

The weighted Cohen κ coefficient (using linear weights) was calculated to check consistency between the scores of the two radiologists and interpreted as in [20]. For each parameter, the mean and standard deviation were calculated. For further analysis, the scores of the two radiologists were averaged. The comparisons between different acceleration or reconstruction techniques for the new dataset were performed using the Wilcoxon signed-rank test. The Wilcoxon–Mann–Whitney test was used to compare relative differences in quality between different datasets; *p*-values < 0.05 were considered statistically significant.

## Results

### Impact of acceleration techniques on MoCo T1-weighted BraCoil images

The detailed results are summarized in Table 1. Images acquired with an acceleration factor of 2 obtained the significantly best scores for rank, overall quality, motion artifacts, and folding artifacts. These images also demonstrated less pronounced motion artifacts.

**Table 1:** Evaluation results of MoCo T1-weighted images acquired with different acceleration techniques

Parallel imaging							Scanning type			
	2-fold	*p*-value (2-fold *versus* 3-fold)	3-fold	*p*-value (3-fold *versus* 4-fold)	4-fold	κ	Standard	*p*-value (standard *versus* elliptical)	Elliptical	κ
Acquisition time (s)	123	–	89	–	76		123	–	82	–
Rank	1.27 ± 0.39	0.01*	2.12 ± 0.55	0.08	2.62 ± 0.42	0.35	1.42 ± 0.34	0.69	1.58 ± 0.34	-0.08
Overall quality	3.62 ± 0.55	0.03*	2.81 ± 0.69	0.03*	2.12 ± 0.68	0.18	3.85 ± 0.43	1	3.81 ± 0.48	-0.02
Motion artifacts	3.65 ± 0.43	0.01*	2.85 ± 0.72	0.07	2.27 ± 0.67	0.23	–	–	–	–
Folding artifacts	3.81 ± 0.48	0.003*	2.88 ± 0.46	0.001*	2.08 ± 0.34	0.25	–	–	–	–

Scores are presented as mean ± standard deviation of the scores of the two radiologists. The results demonstrate that elliptical scanning does not reduce image quality

Further analysis of the retained 2-fold accelerated images revealed no statistically significant differences between elliptical scanning and full Cartesian acquisition. The statistical analysis conducted independently for each radiologist also did not reveal any significant difference between the scanning types. The duration of the T1w sequence with 2-fold acceleration is reduced by 33% from 123 to 82 s with elliptical scanning.

### Comparison between prone and supine images with and without motion correction

Detailed results for the evaluation of the retained images with 2-fold acceleration and elliptical scanning are presented in Table 2. Significant differences between MoCo supine data and prone data were found for folding and motion artifacts and uniformity in T1-weighted images, and overall quality, sharpness, and motion artifacts in T2-weighted images. No significant difference in the overall quality of T1-weighted prone and supine MoCo images was revealed. Mean image quality was better after motion correction in almost all metrics, although not statistically significant.

**Table 2:** Image quality scores for prone images and supine images without correction or GRICS MoCo reconstruction

Sequence	Metric	Supine no MoCo	*p*-value (MoCo *versus* no MoCo)	Supine with Moco	*p*-value (supine with MoCo *versus* prone)	Prone	κ
T1-weighted	Overall quality	2.70 ± 0.79	0.371	2.95 ± 1.04	0.063	3.70 ± 0.26	0.41
	Sharpness	3.30 ± 0.59	1.000	3.25 ± 0.75	0.125	3.65 ± 0.41	0.29
	Motion artifacts	3.10 ± 0.66	0.125	3.45 ± 0.60	0.002*	4.55 ± 0.44	0.29
	Folding artifacts	3.00 ± 0.67	0.195	3.35 ± 0.85	0.008*	4.25 ± 0.68	0.48
	Chemical artifacts	4.90 ± 0.21	1.000	4.90 ± 0.21	0.500	5.00 ± 0.00	-0.02
	Uniformity	4.15 ± 0.47	0.063	3.85 ± 0.47	0.016*	4.45 ± 0.16	-0.07
T2-weighted	Overall quality	4.05 ± 0.60	0.188	4.30 ± 0.42	0.008*	4.90 ± 0.21	0.41
	Sharpness	4.05 ± 0.50	0.359	4.25 ± 0.26	0.008*	4.80 ± 0.26	0.29
	Motion artifacts	4.40 ± 0.46	0.313	4.60 ± 0.32	0.016*	5.00 ± 0.00	0.29
	Folding artifacts	4.65 ± 0.41	0.438	4.85 ± 0.34	0.500	5.00 ± 0.00	0.48
	Chemical artifacts	4.70 ± 0.26	1.000	4.70 ± 0.26	0.500	4.55 ± 0.28	-0.02
	Uniformity	4.55 ± 0.28	1.000	4.55 ± 0.28	0.063	4.85 ± 0.24	-0.07

Scores are presented as the means of the scores of the two radiologists. * Statistical significance

### Comparison of supine data with the BraCoil *versus* the Body 18 coil

The inter-study analysis (Table 3) showed that the overall quality of the images without motion correction was closer to the prone acquisition with the standard coil when using the BraCoil instead of the Body 18 coil.

**Table 3:** Comparison of the relative image quality difference (Δ_relative_) to prone acquisition for supine acquisition with Body18 and BraCoil with and without motion correction

Sequence	Motion correction	Body 18 coil (refs. 12 and 13)	*p*-value (BraCoil *versus* Body18 coil)	BraCoil (this study)
T1-weighted	No	81 ± 29% [13]	0.009*	33 ± 39%
	Yes	74 ± 15% [13]	0.020*	27 ± 39%
T2-weighted	No	57 ± 31% [12, 13]	0.004*	18 ± 15%
	Yes	15 ± 20% [12, 13]	0.339	18 ± 9%

Lower percentages indicate a lower difference to the prone images, *i.e*., better image quality. Acquiring the images with BraCoil led to significantly better quality in most cases

* Statistical significance

**Fig. 1:**
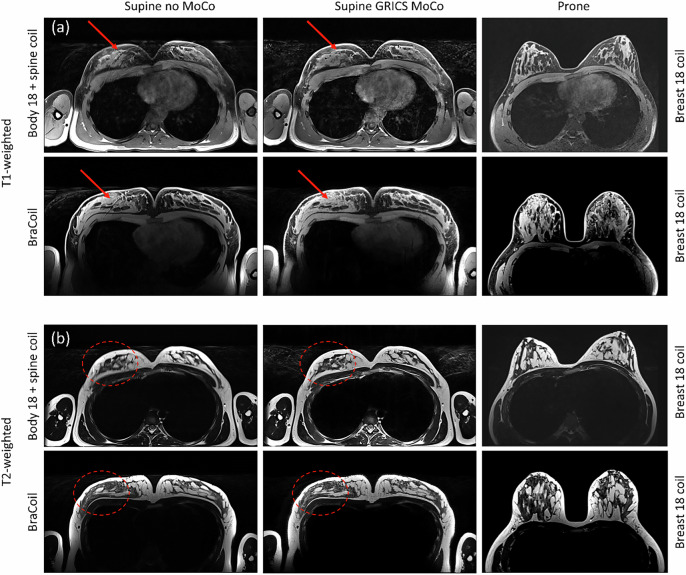
**a** T1- and (**b**) T2-weighted images with BraCoil *versus* Body18 coil with (central column) and without (left column) motion correction (MoCo). The right column shows the corresponding prone images for comparison. Prone images have been zero-padded for aesthetic purposes. The coils used for the supine position are indicated on the left, and the coil used for the prone position is noted on the right. The red arrows point to the ghosting artifacts corrected by MoCO, and the red dashed ellipses indicate the regions where the sharpness was improved after the motion correction

Examples of images acquired using the Body18 coil (subject S3 from [14]) and the BraCoil (subject S10 from the current study) are presented in Fig. 1.

## Discussion

We present a methodological improvement for supine breast MRI. Although it may be less well tolerated by claustrophobic patients, this position is feasible for patients with colostomy or during pregnancy, and improves patient comfort [21]. Additionally, this positioning is better aligned with ultrasound [22] and surgical conditions, though some differences remain, particularly in arm placement.

This study allowed the selection of suitable acceleration techniques for MoCo supine breast MRI using the dedicated wearable BraCoil. Notably, it revealed that elliptical scanning could be used without detriment to image quality and save a considerable amount of acquisition time. This allowed scanning to remain within the recommended 90-s acquisition time limit [23]. This limit was not respected in our previous studies (123 s) [13, 14], and it is not reachable using other motion management techniques, such as classic respiratory gating (120–540 s) [24] or the ZMART technique (190 s) [25]. It is important to note that employing different reconstruction techniques may result in a different selection of acceleration techniques. Notably, achieving higher acceleration factors may be possible if the reconstruction problem is less ill-posed (*e.g*., without motion correction).

Comparisons with previous studies revealed a general improvement over the Body18 coil. This improvement was significant for T1-weighted images, and more pronounced when no motion correction was applied for both T1- and T2-weighted images. This improvement of images without motion correction is likely due to BraCoil’s chest-conforming design, which may reduce the respiratory motion amplitude. Also, it is possible that GRICS reconstruction may correct BraCoil images less efficiently than that acquired with the standard torso coil, given the impossibility of installing motion sensors directly on the chest due to the form-fitting design of the BraCoil, as discussed by Isaieva eta al. [17]. Using a sensor installable closer to the region of interest (*e.g*., optical accelerometer) or enhancing the GRICS algorithm using artificial intelligence [26] might further improve its performance.

Limitations of our study include the small sample size, the single-center design, and the subjective nature of image quality assessment in healthy volunteers. It should also be noted that the radiologists were often able to identify the position (prone or supine) from the image’s appearance. Inter-rater variability was more pronounced where differences between images were subtle (*e.g*., elliptical scanning, chemical shift artifacts) and in assessing uniformity, which is particularly subjective. We expect that the inclusion of pathological cases will lead to more robust radiological scoring.

In summary, in the supine position, the use of the BraCoil generally improves the quality of MoCo breast MRI compared with the Body18 coil used in previous studies, demonstrating its potential to approach the quality of prone acquisitions. The GRICS motion correction technique did not provide significant benefits for BraCoil acquisitions in this study. The combination of the BraCoil and GRICS motion correction has narrowed the gap between supine and prone imaging, advancing high-quality breast MRI in a position better aligned with other modalities. The next phase of this study will involve breast cancer patients to assess differences in diagnostic image quality.

## Data Availability

The experimental data, collected from volunteers, are available upon a reasonable request.

## Abbreviations

GRICS: Generalized reconstruction by inversion of coupled systems
MRI: Magnetic resonance imaging
